# Exosomes isolation and characterization in serum is feasible in non-small cell lung cancer patients: critical analysis of evidence and potential role in clinical practice

**DOI:** 10.18632/oncotarget.7638

**Published:** 2016-02-23

**Authors:** Simona Taverna, Marco Giallombardo, Ignacio Gil-Bazo, Anna Paola Carreca, Marta Castiglia, Jorge Chacártegui, Antonio Araujo, Riccardo Alessandro, Patrick Pauwels, Marc Peeters, Christian Rolfo

**Affiliations:** ^1^ Department of Biopathology and Medical Biotechnology, Section of Biology and Genetics, University of Palermo, Palermo, Italy; ^2^ Institute of Biomedicine and Molecular Immunology (IBIM), National Research Council, Palermo, Italy; ^3^ Phase I-Early Clinical Trials Unit, Oncology Department, Antwerp University Hospital (UZA) and Center for Oncological Research (CORE) Antwerp University, Wilrijkstraat, Edegem, Antwerp, Belgium; ^4^ Department of Oncology, Clínica Universidad de Navarra, Pamplona, Spain; ^5^ Service of Medical Oncology, Centro Hospitalar do Porto, Instituto de Ciências Biomédicas Abel Salazar, University of Porto, Porto, Portugal; ^6^ Molecular Pathology, Pathology Department, Antwerp University Hospital (UZA) and Center for Oncological Research (CORE) Antwerp University, Wilrijkstraat, Edegem, Antwerp, Belgium; ^7^ Oncology Department, Antwerp University Hospital (UZA) and Center for Oncological Research (CORE) Antwerp University, Wilrijkstraat, Edegem, Antwerp, Belgium

**Keywords:** exosomes, NSCLC, liquid biopsies, biomarkers, microRNAs

## Abstract

Exosomes are nano-sized vesicles of endolysosomal origin, released by several cytotypes in physiological and pathological conditions. Tumor derived exosomes, interacting with other cells of the tumor microenvironment, modulate tumor progression, angiogenic switch, metastasis, and immune escape. Recently, extracellular vesicles were proposed as excellent biomarkers for disease monitoring and prognosis in cancer patients. Non-small cell lung cancer (NSCLC) has a poor 5-year survival rate due to the delay in the detection of the disease. The majority of patients are diagnosed in an advanced disease stage. Exosomes might be promising beneficial tools as biomarker candidates in the scenario of NSCLC, since they contain both, proteins and miRNAs. The clinical case reported in this manuscript is a proof of concept revealing that NSCLC exosomes and sorted miRNAs might constitute, in a near future, novel biomarkers. This review summarizes the role of exosomes in NSCLC, focusing on the importance of exosomal microRNAs in lung cancer diagnosis and prognosis.

## INTRODUCTION

Biomarkers constitute one of the most studied tools to understand and diagnose malignancies and to predict the outcome of cancer patients. A valuable biomarker is required to be objectively measured and evaluated as an indicator of physiological or pathological processes or pharmacological responses to a specified therapeutic strategy [1].

Extracellular vesicles (EVs) have recently raised a considerable interest in the field of biomarker discovery. A relevant feature of EV-based biomarker analysis is the significant reduction in complexity of the sample when compared to whole body fluids [2].

Extracellular vesicles (EVs) are released by several cytotypes, constitutively and/or after cell activation. Recently, extracellular vesicles have been classified in different populations, differing in their molecular composition and subcellular origin. The two populations of vesicles most studied and characterized are microvesicles and exosomes.

The term exosome was coined by Johnstone *et al* [3] in a study to understand the biologic process underlying the differentiation of reticulocytes to mature erythrocytes. The authors observed that maturing reticulocytes contained large citoplasmatic invaginations filled with small membrane vesicles of uniform size (40-100 nm). These vesicles were released in the extracellular space in order to eliminate transferrin receptor. After ten years, Raposo *et al*. showed that Epstein-Barr virus (EBV)-transformed B-lymphocytes are able to secrete exosomes containing molecules that are essential for the adaptive immune response, specifically, dimers of the Major Histocompatibility Complex class II (MHCII) bound to antigenic peptides to be presented to T cells [4]. These vesicles contain a tissue-specific signature composed of proteins and selectively packaged RNAs. Patients with cancer show higher concentrations of exosomes in blood and moreover, these exosomes carry tumor-specific molecules, such as DNA and their specific gene mutations [5]. Therefore, exosomes might become true predictive and prognostic biomarkers. More specifically in lung cancer, exosomes will be able to identify different subpopulations of patients according to their molecular characteristics that may be treated with selective targeted therapy.

## NON-SMALL CELL LUNG CANCER

A poorly early detection, associated with limited efficacy of the treatments for advanced disease, is responsible for the low survival rates in NSCLC patients [6].

The development of new diagnostic, prognostic and predictive markers could significantly enhance its early detection outcome. Results from the Collaborative Advanced Stage Tissue Cancer (CASTLE) network are particularly awaited. In fact, this study aims to collect tumor, plasma and serum samples from stage IV NSCLC patients before treatment to test a panel of biomarkers in order to predict response (NCT01574300). Early detection is particularly crucial for tumors without clinical symptoms during the initial stages of their development, such as lung cancer. Stage IIIB/IV NSCLC patients have a poor prognosis and the majority of them, not presenting a drugable molecular alteration, have a median survival around 12 months using platinum based chemotherapy doublets [7]. The era of molecular targeted therapy in lung cancer started in 2004, when activating mutations in the epidermal growth factor receptor (EGFR) and their correlation with clinical response to EGFR tyrosine kinase inhibitors (TKIs) were discovered [8].

The epidermal growth factor receptor (EGFR), also named HER1, is the cell-surface receptor for extracellular protein ligands that are members of the epidermal growth factor family (EGF-family). The *EGFR* activating mutations are able to confirm sensitivity to EGFR-TKIs. Several randomized clinical trials have shown, both in Caucasian and Asian population, superior overall response rates (ORRs) and progression free survival (PFS) for patients receiving EGFR-TKIs (Erlotinib, Gefitinib, Afatinib), compared to standard chemotherapy in patients with NSCLC harboring tumors with *EGFR* activating mutations [9–11]. *EGFR* mutations in NSCLC are present in the range of 13% to 18% of Caucasian patients, and in about 40% of Asian patients [9].

Unfortunately, the majority of *EGFR*-mutant NSCLC patients that show an initial radiological response to EGFR-TKIs, eventually progress and their tumors develop different mechanisms of resistance [12]. One most frequent known mechanism of resistance to EGFR-TKIs is associated with the appearance of a single recurrent missense mutation, T790M, within the EGFR kinase domain [12]. The presence of this mutation can be also detected ex novo [13, 14]. The mutation is found in the methionine residue at position 790, causing a steric hindrance in the interaction with the inhibitor, preventing its binding to the EGFR kinase domain that still preserves catalytic activity [15, 16]. Several strategies are ongoing in order to overcome these resistance mechanisms, including third generation EGFR-TKIs, like AZD9291 [17].

A similar scenario occurs for other NSCLC predictive biomarkers. Specifically, the *EML4-ALK* fusion gene is a predictive biomarker for response to first generation ALK-TKIs [18], presenting a significant clinical benefit when used in first-line treatment compared with chemotherapy for *EML4-ALK* translocated tumors in NSCLC patients [19, 20].

Not with standing, the recent emergent knowledge on the role of different predictive and prognostic biomarkers makes the molecular analysis of the tissue mandatory. Due to the small size and the remote localization of many NSCLC tumors, obtaining tissue for further analysis is a tricky task. Currently, several efforts are ongoing in order to obtain all this biological information through non-invasive means. Circulating tumor cells (CTCs), circulating tumor DNA (ctDNA) and exosomes, all different components of the commonly used concept “liquid biopsies”, are part of this answer [21].

Through new sensitive and faster technologies for nucleic acids analysis, such as digital droplet PCR (ddPCR) coupled with Next Generation Sequencing (NGS) technologies, it was possible to detect and sequence ctDNA and CTCs genes in order to exploit them as new source of biological informations, since both these liquid biopsy components mirrors the genetic and epigenetic status of the tumor mass [22–24].

The detection of CTCs in the blood of cancer patients has been usually related to metastatic stages [25, 26]. However, with these new sensitive technologies, it has been demonstrated that CTCs could be detected also in early stages of several cancer types, such as in lung cancer, thus enhancing their role as predictive and prognostic biomarkers [27–29]. Recently, CellSearch® system was approved from FDA as first tool for CTCs analysis in order to have another prognostic biomarker source on metastatic breast cancer [30].

Moreover, it has been demonstrated that is feasible to characterize proteomic and miRNA profiling of exosomes. Interestingly enough, miRNAs present in the exosomes, have been correlated with the presence of some specific molecular alterations, such as *EGFR* mutations, among others [31].

## EXOSOMES IN CANCER PATIENTS: ORIGIN AND POTENTIAL CLINICAL INTEREST

Exosomes are spherical nano-sized vesicles that represent a distinct class of membrane vesicles, with a density of 1.13-1.19 g/ml and a diameter of 40-100 nm. These vesicles contain a high number of molecular cargo such as mRNA, miRNAs, proteins and DNA protected by a lipid bilayer.

Exosomes have an endocytic origin and are released by different cell types in both normal and pathological conditions [32]. The exosomal composition mirrors the parental cell, but several proteins, such as CD63, CD81, CD9, TSG-101, ALIX, and HSP70, are contained in the exosomes released by all cytotypes. These proteins are considered exosomal markers and they are commonly used to identify the presence of vesicles as true exosomes [33].

**Figure 1:**
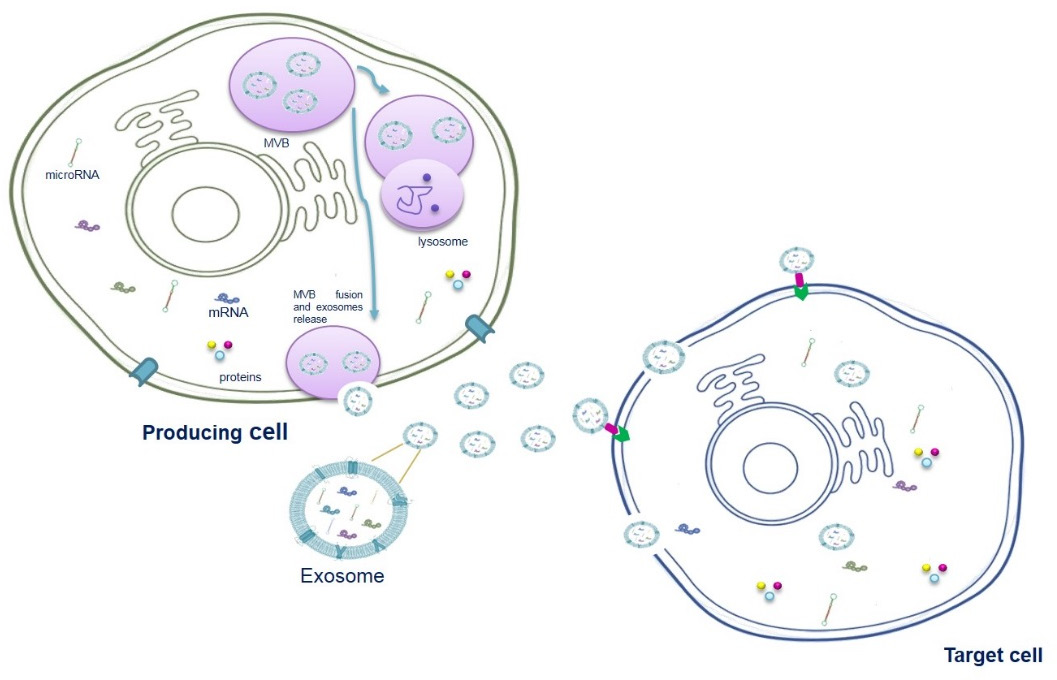
Schematic model of exosome origin and release Exosomes derived from the intraluminal vesicles (ILVs) of multivesicular bodies (MVBs), formed through the reversed budding of the membrane of late endosomes. ILVs encapsulate material from cytoplasm including RNA (both mRNAs and miRNAs) and proteins. Multivesicular bodies can fuse with lysosomes to degrade their content or fuse with the plasma membrane to release the ILVs into the extracellular space.

Current models suggest that exosomes are formed within the endocytic pathway and released from the plasma membrane *via* multivesicular bodies (MVBs). MVBs are formed from early endosome maturation, and they can fuse with lysosomes or the plasma membrane. When MVBs fuse with lysosomes, their content will undergo lysosomal degradation. The fusion of MVBs with the plasma membrane induces the release of intraluminal vesicles (exosomes) into the extracellular space (Figure 1). Exosome formation needs the endosomal sorting complex required for transport (ESCRT) [33].

Several studies hypothesize a pleiotropic role for tumor derived exosomes; they may affect the growth and survival of tumor cells, degradation of the extracellular matrix, stromal remodeling, angiogenesis and drug resistance as well as modulation of the immune system [34].

In this field, the exosomes released from macrophage and NK cells may regulate the tumor invasiveness and its metastatic ability and may have a role in immune cytotoxicity, respectively [35]. It has also been described that mast cell-derived exosomes contain mRNAs and miRNAs with biological activity, immunological proteins (such as MHC-II, CD86, CD40, CD40L, FcƐRI subunits alpha and beta) and could be involved in B-cell activation, inflammation process and immunomodulation [36–38]. Extracellular vesicles can also carry drugs and represent a promising strategy for drug delivery. Interestingly, proteins differentially expressed in extracellular vesicles, can be used as biomarkers for early detection, diagnosis and prognosis of cancer [21].

**Figure 2:**
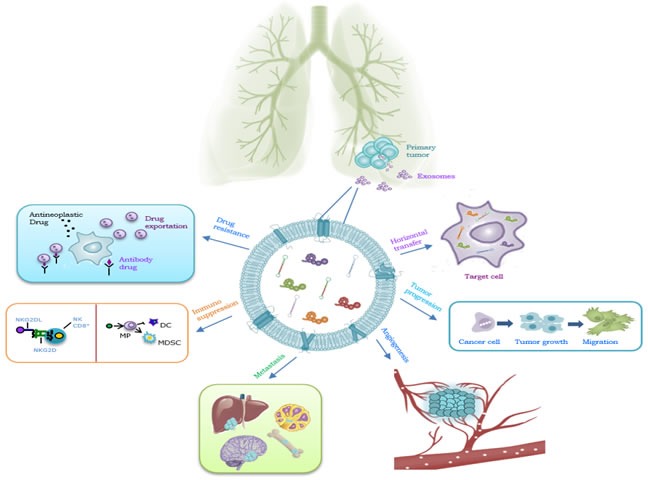
Role of exosomes in NSCLC Exosomes have a key role in : 1 horizontal transfer of mRNAs and miRNAs from cancer cells to cells of microenvironment; 2 tumor progression, inducing cells motility; 3 angiogenesis; 4: metastatization; 5: immunosuppression; 6: drug resistance.

Specially relevant is the role of extracellular vesicles in transferring information between cells. That information may be crucial for tumor progression and angiogenesis according to a recent published research showing the involvement of cancer cells-released exosomes in that matter [39]. In fact, several studies support that exosomes may play a relevant role in remodeling the tumor microenvironment and in tumor progression *via* an enhanced angiogenesis and the preparation of the metastatic niche (Figure 2). More specifically, melanoma-derived exosomes have been shown to promote metastasis through the preparation of the metastatic niche by the activation of bone marrow-derived progenitor cells [40]. Exosomes have been shown to be involved in several cellular functions and intrinsic mechanisms of cancer where they possibly constitute valuable biomarkers [21].

Interestingly enough, although exosomes have been better characterized in peripheral blood samples, they can be isolated as well from most body fluids, including urine, amniotic fluid, serum, pleural effusion, saliva, breast-milk, cerebrospinal fluid, and nasal secretions [41].

As previously mentioned, the components of exosomes are varied and include proteins, lipids and different subtypes of RNA, which can be consulted in the ‘Exosome Content Database’ (www.exocarta.org) [42]. Recently, doubled-stranded DNA has been also discovered in exosomes opening a new perspective in this field [43].

Currently, the Exosome Content Database contains about 4,563 proteins, 1,639 mRNAs, 764 miRNAs and 194 lipids. Among the most frequently, identified proteins in exosomes are membrane transport and fusion proteins, heat-shock proteins, GTPases, MVB biogenesis proteins, cytoskeletal proteins, metabolic enzymes, signal transduction proteins and carriers. Thanks to proteomic analysis, more than 4,400 different proteins have been detected by the use of mass spectrometry. Certainly, the specific protein composition depends on the cell type or tissue source [42, 44].

Exosomes are also enriched in lipids that may act as important signaling molecules. They include cholesterol, diglycerides, sphingolipids, phospholipids, glycerophospholipids and phosphatidylserine, which have a role in exosomes recognition and internalization [45]. Exosomes are also enriched in bioactive lipids, such as prostaglandins and leukotrienes [46].

In 2007 Valadi *et al*. discovered exosomal miRNAs [47], a year after Taylor *et al*. reported that eight miRNAs, previously demonstrated as diagnostic markers for ovarian cancer might be identified in biopsies of ovarian cancer as well as contained in serum exosomes isolated from the same patients. These exosomal miRNAs were not detectable in normal controls, suggesting that they are easily attainable and may be potentially used as diagnostic markers, a worthy alternative for biopsy profiling [48]. miRNAs are short single-stranded, endogenous, non-coding RNA molecules that are involved in binding partial complementary sequences within the 3′-UTR of the target mRNAs. Global gene expression profiles have revealed numerous miRNAs that were deregulated in cancer compared with normal tissues [48]. The miRNAs associated with oncogenesis are also termed “oncomirs”. Depending on the main target, oncomirs are generally classified as tumor suppressive and oncogenic miRNAs where, in general, tumor suppressive molecules repress protein-coding oncogenes and oncogenic miRNAs that repress protein-coding tumor suppressors. The profile of miRNAs found in exosomes is rather specific, since particular repertories of miRNAs are selectively sorted into exosomes, while other miRNAs are usually excluded. As a good example of this important role, our group recently demonstrated, *in vitro* and *in vivo*, a selective sorting of the oncomir miR-21 in exosomes released by chronic myelogenous leukemia cell lines after curcumin treatment [49]. Furthermore, unlike other subtypes of RNA [47], exosomal miRNA profile is similar to the miRNA profile of parental cancer cells, increasing the interest of scientists in the use of exosomal miRNA for diagnosis of cancer [50, 51]. Moreover, the regulatory properties attributed to tumor-derived exosomes are essential in shaping the tumor microenvironment and promoting tumor growth and metastasis [52, 53].

Clear examples of potential clinical applications can be found in melanoma patients, in which released exosomes were demonstrated to contain high levels of the proteins Caveolin-1 and CD63 [54]. In addition, in glioblastoma patients, exosomes have been described as an amenable biomarker for the identification of disease-specific molecules [55]. Que *et al*. also demonstrated that miR-17-5p and miR-21 were highly expressed in exosomes isolated from patients with pancreatic cancer (PC). On the one hand, serum exosomal miR-17-5p was higher in PC patients than in non-PC individuals (with ampullary carcinoma, benign pancreatic tumors or chronic pancreatitis) and healthy donors. High levels of miR-17-5p were correlated with the presence of metastasis and advanced PC stage. On the other hand, the serum exosomal miR-21 levels in PC were higher than in cohorts of healthy donors and in patients with chronic pancreatitis, but they were not correlated with PC differentiation or tumor stage [56].

Serum levels of miR-141 and miR-375 are shown to be correlated with tumor progression in prostate cancer [57], showing to be valuable markers in the diagnosis of this tumor type. Moreover, Tanaka *et al*. showed that exosomal miR-21 expression is upregulated in serum from patients with esophageal squamous cell cancer (ESCC) *versus* serum from patients with benign diseases, without systemic inflammation. In that case, exosomal miR-21 was positively correlated with tumor progression and aggressiveness, suggesting that this miRNA might be a useful target for cancer therapy [58].

Chiba *et al*. also indicated that exosomes derived from three colorectal cancer (CRC) cell lines contained both mRNAs miRNAs, and were delivered into HepG2 and A549 cells. These novel findings indicate that exosomes, containing several RNAs, shuttle between different cell types, and most probably may regulate gene expression into the recipient cells [59].

## MOLECULAR COMPONENTS OF EXOSOMES IN NSCLC

As previously described, the molecular profile in lung cancer, specifically in NSCLC, plays a crucial role in prognosis and prediction of the outcome of our patients. The idea to identify specific molecular patterns in exosomes isolated from NSCLC patients results very attractive. Several exosomal components have been studied. In this section, we will accurately describe the two more relevant ones, proteins and miRNAs.

## PROTEINS

NSCLC exosomes contain several tumor-associated proteins, EGFR, KRAS, extracellular matrix metalloproteinase inducer (EMMPRIN), claudins and RAB-family proteins. EGFR is overexpressed in several human cancers and its overexpression correlates with poor prognosis in a large number of malignancies, including NSCLC. Exosomal mediated transfer of oncogenic EGFR from human squamous cell carcinoma to tumor-associated endothelial cells was shown to activate MAPK and AKT cell signaling pathways and to promote endothelial VEGF expression [60]. In order to study EGFR-containing exosomes in NSCLC, Huang *et al* [61] compared the exosomes content between tumor biopsies from NSCLC patients with chronic inflammation lung tissues. Eighty percent of the exosomes isolated from those NSCLC samples was positive for surface EGFR by immune staining compared to 2% of the exosomes in chronic inflammatory lung tissue.

The possibility to determine the presence of *EGFR* mutations in exosomes could represent a real innovation in the development of exosomes as clinically useful liquid biomarkers, constituting a reliable tool for diagnosis and treatment selection. EGFR incorporated in exosomes could generate tollerogenic dendritic cells and Treg cells, which lead to the suppression of CD8 cells [61]. Exosomes carry proteins that may not only predict the outcomes of patients, but may actively participate in tumorigenesis *via* immune system deregulation, as well. Therefore, although exosomes may also play an important role in immune system regulation, its potential activity against cancer, by supporting immunotherapy, remains unknown.

Regarding the potential translation of exosomes into NSCLC diagnostic characterization, Jakobsen *et al* demonstrated that exosomal proteins are potential diagnostic markers in advanced NSCLC. Plasma isolated from 109 NSCLC patients with advanced stage (IIIa-IV) disease and 110 matched control subjects was analyzed with an Extracellular Vesicle Array (EV Array). This array, used to phenotype exosomes, contained 37 antibodies targeting lung cancer-related proteins and was used to capture exosomes. EV Array constitutes a tool to get information on exosomal content with very limited sample requirement and which can be optimized and adjusted to fit individual sets of samples [62].

Exosomal proteins may well reflect pathological processes associated with the disease. Since some of those proteins may play a critical role in tumorigenesis they might constitute good diagnostic and prognostic biomarkers in NSCLC [63].

The idea of obtaining proteomic information in lung cancer, from other body fluids beyond blood samples, led to Li *et al* [64] to investigate this extent in urine samples. In their experiment, urine samples from eight chemo-naïve NSCLC patients were compared with samples from ten healthy volunteers. Interestingly enough, the analysis showed that NSCLC patients were able to release exosomes in urine. Moreover, high expression of leucine-rich α-2-glycoprotein (LRG1) measured by immunohistochemistry in primary tumor, showed a positive correlation rate of 65% with LRG1 presence in urine samples, suggesting that LRG1 in urinary exosomes might be derived from primary tumor tissue. These authors concluded that urinary LRG1 may be a candidate biomarker for non-invasive diagnosis of NSCLC in selected individuals [64].

Other potential clinical use of the study of exosomes in lung cancer patients relays on their suspected role in the prediction of treatment resistance. For unselected lung cancer populations, platinum doublet chemotherapy remains the most beneficial treatment for patients with advanced NSCLC. A recent preclinical study on exosomes and DNA-damaging Platinum (DDP), an agent that may cause interstrand and/or intrastrand crosslinks in the DNA of tumor cells, showed that when A549 cells are exposed to DDP, the expression levels of several miRNAs and mRNAs, usually associated with DDP sensitivity, drastically change in exosomes, probably mediating the DDP resistance of A549 cells. Exosomes released by A549 cells during DDP exposure decreased the sensitivity of other A549 cells to DDP, which may be mediated by miRNAs and mRNAs exchange by exosomes *via* cell-to-cell communication [65].

In summary, there are different potential clinical applications of exosomal proteins in NSCLC as well as in other malignancies as diagnostic and predictive biomarkers.

## MICRORNAS

An important number of clinical trials currently use miRNAs profiling [66], in fact, miRNA-based therapeutic approaches are being developed by several pharmaceutical companies.

Recent findings revealed the important regulatory roles of miRNAs in a complex multistep process of invasion-metastasis. Overexpression of miR-126 reduced NSCLC cell adhesion, migration, and invasion, which may be partially due to the regulation of adaptor protein Crk, also known as p38 or proto-oncogene c-Crk [67].

The expression of miR-34a in the H1299 NSCLC cell line resulted in massive apoptosis and exogenous delivery of lipid formulated miR-34a reduced tumor size in a mouse model of NSCLC, suggesting its possible therapeutic potential [68].

An overexpression of 12 specific miRNAs (Table 1) in NSCLC patients tissue compared to normal lung tissue was demonstrated by Yanaihara *et al* [69], indicating a potential diagnostic miRNA signature. Among these, selected miRNAs, miR-146a, targeting EGFR, is differentially expressed in various cancer histologies. Preclinical experiments performed by this group, in five different NSCLC cell lines, revealed the miR146a-dependent suppression of cell growth, induction of apoptosis, inhibition of cell migration, and suppression of EGFR downstream signaling effectors. Surprisingly, miR-146a was also able to enhance the inhibition of cell proliferation induced by drugs targeting the EGFR. These effects were independent of the *EGFR* mutation status. miR-146a was also shown to be generally downregulated in lung cancer patients and low levels of this miRNAs correlated with the presence of metastasis [69].

**Table 1:** Selected overexpressed microRNAs discovered in NSCLC

miR-17-3p
miR-21
miR-106a
miR-146
miR-155
miR-191
miR-192
miR-203
miR-205
miR-210
miR-212
miR-214

Recently, Rodriguez *et al*. conducted a prospective analysis of blood and bronchoalveolar lavage (BAL) samples from 30 NSCLC patients and 75 patients without oncological diseases. They found that exosomes and miRNAs levels were higher in both plasma and BAL from NSCLC patients. The authors revealed that miR-126 was only determinated in plasma samples [70]. MiR-126 inhibits the NSCLC cells proliferation *via* EGF-like domain-containing protein 7 (EGFL7) and targets Sdf-1 cytokine to reduce the recruitment of mesenchymal stem cells and inflammatory monocytes to primary tumors, inhibiting lung metastasis appearance [71]. In another study, five determined miRNAs were selected from a panel of 365 miRNAs in the plasma of 28 NSCLC patients and 20 healthy controls. A validation of these five selected miRNAs (let7f, miR-20b, miR-30e-3p, miR-223and miR-301) was independently performed by real-time PCR in plasma from 78 NSCLC and 48 controls, and correlated with pathologic parameters and survival. Levels of let-7f, miR-20b and miR-30e-3p were decreased in plasma vesicles of NSCLC patients. The levels of let-7f and miR-30e-3p was able to discriminate between two groups of patients for stage of disease and therefore their surgical options. More interestingly, plasma levels of miR-30e-3p and let-7f in NSCLC patients has been also associated with poor clinical outcome [72].

The miRNA profile in exosomes is similar to the primary tumor profile and this feature may be a powerful tool in different aspects such as early diagnosis, prognosis and prediction of response.

After having discussed the role of exosomes in the biology of tumor progression and their potential to translate the preclinical experience into clinical practice, a clinical case followed by our group will be described and discussed. With this case, we would like to demonstrate that isolation and characterization of exosomes in patients with NSCLC is feasible in a daily practice.

## CLINICAL CASE

We present a case of a 70 years old woman with no relevant past medical history diagnosed with stage IV NSCLC, (adenocarcinoma histotype) and harboring an *EGFR* activating mutation by a deletion in exon 19 (c.2236_2250del15, p.Glu74_Ala750del). The patient was treated with Gefitinib 250 mg/daily. After two months of treatment, a computerized tomography (CT) scan showed a partial response in the primary tumor. The patient continued on treatment for a total of 10 months with stabilization of disease and an improvement in quality of life.

Before the start the treatment with Gefitinib, a blood sample was collected and plasma was separated. The plasma sample was processed with a commercial kit (Invitrogen™, Carlsbad) to collect exosomes. In order to characterize the exosomes, we performed a morphological and biochemical analysis. We confirmed exosomes characterization by performing Transmission Electron Microscopy (TEM) analysis, in order to show that the purified vesicles of our patient sample had an average size in the range of the exosomal diameter, between 40-100nm, what is in agreement with data from literature [39].

**Figure 3:**
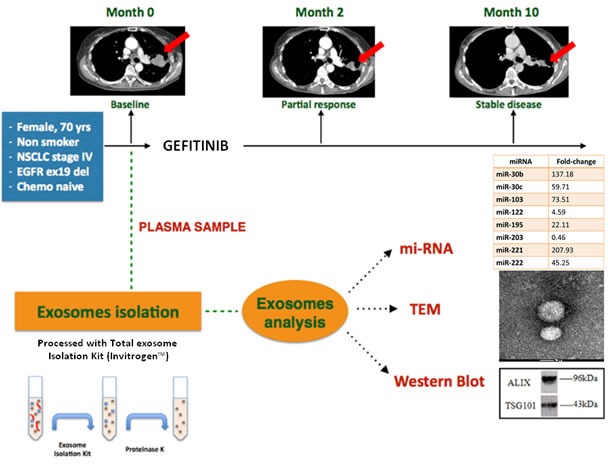
Summary of clinical case report The upper part of the figure shows the computerized tomography evolution. The patient achieved a partial response in 2 months, disease has been stable for 8 more months. The lower part shows a short scheme of exosome isolation and the morphological and biochemical analysis of the exosomes collected by the plasma of NSCLC patient. The miRNAs expression fold-change, relative to the baseline timepoint, is related to healthy control samples.

Vesicles were also analysed by Western blotting using specific antibodies for Alix and TSG-101, both well-known exosomal markers. These data confirmed that the vesicles that were purified from plasma of our patient were indeed exosomes (Figure 3).

Based on literature, we selected eight miRNAs, with a documented role in NSCLC [31]. This panel of miRNAs was analyzed by TaqMan® Real-time PCR [58]. Relative changes in miRNA expression between healthy control and patient samples were determined with the ΔΔCt method; miR-1228-3p was selected as stable endogenous control.

As shown in Figure 3, high levels of miR30b, -30c, -103, -195, -221, -222 were encapsulated in exosomes. These results are concordant with the findings of Garofalo *et al*, indicating that miR-103, −203, −30b-c and −221 −222 have a prognostic value, as a marker, in lung cancer [31]. Yanaihara *et al* [69] also indicated that the expression of 12 selected miRNAs in exosomes from NSCLC patients could play a role in diagnosis, since they were expressed only in exosomes released by NSCLC cells. We found a downregulation of miR-122 and miR-203, possibly predictive of more aggressive and metastatic tumors [31]. Circulating miR-122 was expressed at lower levels in patients with *EGFR* mutated NSCLC compared with wild type *EGFR* NSCLC patients [73]. In concordance with these results, our analysis confirmed a downregulation of this miRNA in exosomes of our *EGFR* mutant NSCLC patient.

With this single case, we show that this exosomal miRNAs profile of our patient is in concordance with the data in the literature. We believe that exosomal miRNAs may have an important role in future diagnostic and prognostic analysis. Our group is currently working in this context, to demonstrate the role of exosomes in monitoring NCSLC patients during the treatment. These observations suggest that NSCLC exosomes and sorted miRNAs may have an important role in diagnosis and prognosis in a near future. An interesting open question regarding the exosomal application in clinical practice it could be the standardization of the correlation between exosomal features and clinical outcomes. In this context, the idea to exploite the correlation between protein and/or miRNA levels of exosome (as part of liquid biopsies) and tumor tissue, it could be extremely attractive.

## DISCUSSION

Throughout this manuscript, we have described the role of exosomes in cancer, principally in lung cancer. Exosomes are small vesicles released by cancer cells to the extracellular medium and are able to shuttle proteins and nucleic acids. Exosomes are involved in several processes such as angiogenesis, premetastatic niche preparation and regulation of tumor immune system responses.

Exosomes carry several validated and surrogate non-invasive biomarkers with diagnostic, prognostic and predictive value. Clinical studies have already described exosomes-associated cancer biomarkers for several cancer types such as prostate, breast and ovarian cancer as well as glioblastoma and melanoma.

The isolation of exosomes and its use in the clinic may substitute invasive procedures for diagnosis or for the follow-up of cancer patients, mainly in NSCLC, where the availability of primary tumor tissue is difficult in most of the patients. The development of novel biomarkers and the establishment of a fluid-based early detection system for lung cancer is crucial to improve the clinical outcome.

This approach brings also positive effects like a reduction of complications derived from tumor biopsies, the possibility to anticipate progression and even the reduction of healthy costs with ambulatory non-invasive techniques. Since the markers must be stable, all these features make exosomes good candidates for become biomarkers in NSCLC. In addition, exosomal proteins and miRNAs are protected from RNase/proteinase-dependent degradation and thus can be stably detected in circulating plasma and serum, making them ideal biomarkers for a number of clinical applications [74]. Another reason to select exosomes as preferable liquid biopsy tool is correlated to their higher concentration in blood of cancer patients [75] in comparison with CTCs, that could lead exosomes to be more exploitable for isolation and analysis methods.

For instance, some biomarkers such as PCA3 and TMPRSS2 are mRNAs not easily detected in body fluids, but are found in exosomes in prostate cancer [76]. Compared with traditional biomarkers, exosomes have other advantages potentially, these vesicles can travel across the endothelium into the circulation allowing serum detection. In contrast to invasive biopsy, exosomes are effective biomarkers in the diversified diagnosis of personalized medicine [77].

For these reasons, our group is focusing on the potential predictive and prognostic value of exosomes collected from NSCLC samples.

The isolation of exosomes from plasma of patients requires specific expertise, because the amount of samples is often insufficient to perform the purification with ultracentrifugation. Recently, the increasing number of commercial kits developed allows researchers to choose the most suitable one for different analysis. In our clinical case, the purification of exosomes with a commercial kit in combination with transmission electron microscopy, Western blotting analysis and miRNAs detection allowed us to demonstrate the presence of exosomes containing miRNAs in plasma of a NSCLC patient and to confirm the data of literature regarding the role of exosomal miRNAs.

Exosomes are also attractive in cancer therapy, as vehicles for administration of antitumor compounds such as anticancer therapies, siRNAs and antigens to target recipient cells [78, 79].

Furthermore, in this field, exosomes derived from immune cells can be used to develop cancer vaccines. Morse *et al* reported a phase I study of dendritic-derived exosomes (dexosomes) immunotherapy in patients with advanced NSCLC. Dexosomes were injected in 13 NSCLC patients for 4 weeks but unfortunately inducing only a weak immune response against the tumor [80]. Viaud *et al* developed a new technology to produce highly immunogenic dexosomes, currently used in a phase II clinical trial to test the clinical benefit of dexosomes to maintain immunotherapy in inoperable NSCLC patients that responded to chemotherapy [81].

These perspectives open infinite possibilities for clinical application. Tremendous multidisciplinary efforts must be done by basic researchers, oncologists and patients, in order to make all this incredible knowledge applicable in a real translational oncology scenario.
